# Complete genome sequence of *Xylanimonas cellulosilytica* type strain (XIL07^T^)

**DOI:** 10.4056/sigs.571102

**Published:** 2010-01-28

**Authors:** Brian Foster, Rüdiger Pukall, Birte Abt, Matt Nolan, Tijana Glavina Del Rio, Feng Chen, Susan Lucas, Hope Tice, Sam Pitluck, Jan-Fang Cheng, Olga Chertkov, Thomas Brettin, Cliff Han, John C. Detter, David Bruce, Lynne Goodwin, Natalia Ivanova, Konstantinos Mavromatis, Amrita Pati, Natalia Mikhailova, Amy Chen, Krishna Palaniappan, Miriam Land, Loren Hauser, Yun-Juan Chang, Cynthia D. Jeffries, Patrick Chain, Manfred Rohde, Markus Göker, Jim Bristow, Jonathan A. Eisen, Victor Markowitz, Philip Hugenholtz, Nikos C. Kyrpides, Hans-Peter Klenk, Alla Lapidus

**Affiliations:** 1 DOE Joint Genome Institute, Walnut Creek, California, USA; 2 DSMZ - German Collection of Microorganisms and Cell Cultures GmbH, Braunschweig, Germany; 3 Los Alamos National Laboratory, Bioscience Division, Los Alamos, New Mexico, USA; 4 Biological Data Management and Technology Center, Lawrence Berkeley National Laboratory, Berkeley, California, USA; 5 Oak Ridge National Laboratory, Oak Ridge, Tennessee, USA; 6 HZI – Helmholtz Centre for Infection Research, Braunschweig, Germany; 7 University of California Davis Genome Center, Davis, California, USA

**Keywords:** Aerobic, Gram-positive, non-motile, cellulases, xylanases, *Promicromonosporaceae*, GEBA

## Abstract

*Xylanimonas cellulosilytica* Rivas *et al*. 2003 is the type species of the genus *Xylanimonas* of the actinobacterial family *Promicromonosporaceae*. The species *X. cellulosilytica* is of interest because of its ability to hydrolyze cellulose and xylan. Here we describe the features of this organism, together with the complete genome sequence, and annotation. This is the first complete genome sequence of a member of the large family *Promicromonosporaceae*, and the 3,831,380 bp long genome (one chromosome plus an 88,604 bp long plasmid) with its 3485 protein-coding and 61 RNA genes is part of the***** G****enomic **** E****ncyclopedia of **** B****acteria and **** A****rchaea * project.

## Introduction

Strain XIL07^T^ (= DSM 15894 = CECT 5975 = JCM 12276) is the type strain of the species *Xylanimonas cellulosilytica* and was first described in 2003 by Rivas *et al*. [1]. It was isolated from a decayed tree, *Ulmus nigra*, in Salamanca, Spain. *X. cellulosilytica* is of high interest because it produces a set of hydrolytic enzymes, cellulases and xylanases that enable the organism to hydrolyze cellulose and xylan. Cellulolytic enzymes from microorganisms have many biotechnological and industrial applications, for example in the food, detergent, paper and textile industries or in the production of biofuels.  Here we present a summary classification and a set of features for *X. cellulosilytica* XIL07^T^, together with the description of the complete genomic sequencing and annotation.

## Classification and features

The most closely related 16S rRNA gene sequences from cultivated strains that are stored in Genbank originate from isolates classified into neighboring genera within the *Promicromonosporaceae*. Among them are some *Isoptericola* species isolated from different habitats, including soil, tufa, decayed wood and the hindgut of the humus-feeding larva of the beetle *Pachnoda ephippiata*. Sequences of 16S rRNAs from several uncultivated bacteria detected in midgut and hindgut of *P. ephippiata* (AJ576375, AJ576390, AJ576391 AJ576404, AJ576378, AJ576417) [2] are apparently the most closely related phylotypes, with 96-97% sequence similarity. Environmental samples from metagenomic surveys do not surpass 92% sequence similarity, indicating that members of the species are not heavily represented in the so far genomically screened habitats (status July 2009).

**Figure 1:**
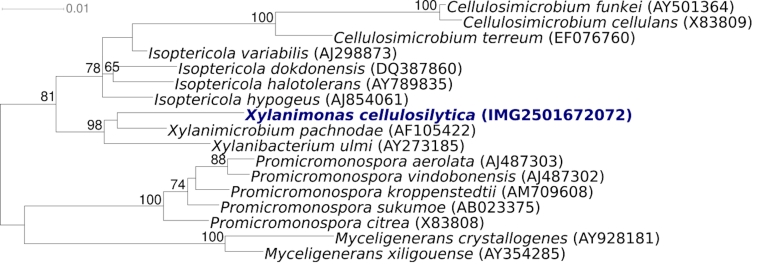
Phylogenetic tree highlighting the position of *X. cellulosilytica* XIL07^T^ relative to the other type strains within the family *Promicromonosporaceae*. The tree was inferred from 1,393 aligned characters [3,4] of the 16S rRNA gene sequence under the maximum likelihood criterion [5] and rooted in accordance with the current taxonomy. The branches are scaled in terms of the expected number of substitutions per site. Numbers above branches are support values from 1,000 bootstrap replicates if larger than 60%. Lineages with type strain genome sequencing projects registered in GOLD [6] are shown in blue, published genomes in bold.

Figure 1 shows the phylogenetic neighborhood of *X. cellulosilytica* XIL07^T^ in a 16S rRNA based tree. The sequences of the three copies of the 16S rRNA gene in the genome differ by up to four nucleotides, and differ by up to five nucleotides from the previously published sequence generated from DSM 15894 (AF403541).

**Figure 2:**
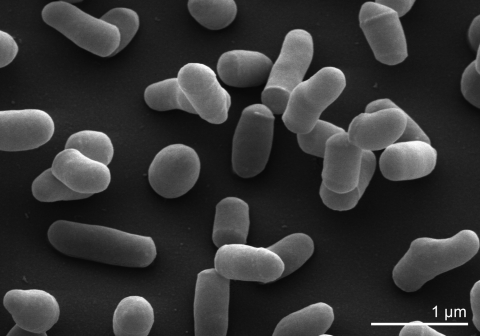
Scanning electron micrograph of *X. cellulosilytica* XIL07^T^

Based on thin section EMs, cells of strain XIL07^T^ were described as coccoid (approximately 1.1 x 0.8 µm) that occur singly [1]. However, SEM images (Figure 2) show coccobacillary forms and short rods, as known from other members of the *Promicromonosporaceae*: *Cellulosimicrobium*, *Isoptericola* and *Promicromonospora *(Table 1). Strain XIL07^T^ is Gram-positive, aerobic or facultatively anaerobic, nonmotile and non-spore-forming. Colonies grown on YED are white-to-cream colored and their morphology is circular, smooth and mostly flat. Strain XIL07^T^ utilizes L-arabinose, carboxymethylcellulose, mannose, maltose, rhamnose, starch and xylan as sole carbon source, and produces acid from amygdalin, L-arabinose, arbutin, cellobiose, fructose, galactose, getobiose, glucose, glycerol, glycogen, lactose, lyxose, maltose, mannose, melozitose, rhamnose, salicin, sucrose, trehalose, turanose and D-xylose. No growth was observed with acetate, citrate, gluconate, inositol, malate or mannitol as carbon sources. Strain XIL07^T^ actively produces amylases, cellulases, gelatinase, xylanases and β-galactosidase and shows weak catalase activity. Esculin was hydrolyzed and nitrate was not reduced [1].

**Table 1:** Classification and general features of *X. cellulosilytica* XIL07^T^ according to the MIGS recommendations [7]

**MIGS ID**	**Property**	**Term**	**Evidence code**
	Classification	Domain *Bacteria*	TAS [8]
		Phylum *Actinobacteria*	TAS [9]
		Class *Actinobacteria*	TAS [10]
		Subclass *Actinobacteridae*	TAS [10]
		Order *Actinomycetales*	TAS [10]
		Suborder *Micrococcineae*	TAS [10]
		Family *Promicromonosporaceae*	TAS [10]
		Genus *Xylanimonas*	TAS [1]
		Species *Xylanimonas cellulosilytica*	TAS [1]
		Type strain XIL07	
	Gram stain	positive	TAS [1]
	Cell shape	coccoid, short rods	TAS [1] & IDA
	Motility	nonmotile	TAS [1]
	Sporulation	nonsporulating	TAS [1]
	Temperature range	mesophile	TAS [1]
	Optimum temperature	30 °C	TAS [1]
	Salinity	not reported	
MIGS-22	Oxygen requirement	aerobic or facultatively anaerobic	TAS [1]
	Carbon source	L-arabinose, carboxymethylcellulose, mannose, maltose, rhamnose, starch and xylan	TAS [1]
	Energy source	chemoorganotroph	TAS [1]
MIGS-6	Habitat	soil	TAS [1]
MIGS-15	Biotic relationship	free living	NAS
MIGS-14	Pathogenicity	non pathogenic	NAS
	Biosafety level	1	TAS [11]
	Isolation	decaying tree	TAS [1]
MIGS-4	Geographic location	Salamanca, Spain	TAS [1]
MIGS-5	Sample collection time	not reported	
MIGS-4.1 MIGS-4.2	Latitude, Longitude	40.965, -5.663	NAS
MIGS-4.3	Depth	not reported	
MIGS-4.4	Altitude	not reported	

Evidence codes - IDA: Inferred from Direct Assay (first time in publication); TAS: Traceable Author Statement (i.e., a direct report exists in the literature); NAS: Non-traceable Author Statement (i.e., not directly observed for the living, isolated sample, but based on a generally accepted property for the species, or anecdotal evidence). These evidence codes are from the Gene Ontology project [12]. If the evidence code is IDA, then the property was directly observed for a living isolate by one of the authors or an expert mentioned in the acknowledgements.

### Chemotaxonomy

The cell wall of *X. cellulosilytica* XIL07^T^ contains A4α-type peptidoglycan (L-Lys-D-Asp). Cell wall sugars are galactose and rhamnose. Mycolic acids are absent. Strain XIL07^T^ contains menaquinone MK-9(H4) as the major respiratory lipoquinone and a lower amount of MK-8(H4). The cellular fatty acid pattern is composed of iso- and anteiso-branched fatty acids with anteiso-C15:0 (12-methyl tetradecanoic acid) being the predominant and iso-C15:0 the minor fatty acid. The major polar lipids are phosphatidylglycerol, diphosphatidylglycerol, phosphatidylinositol, phosphatidylinositol mannosides and other unidentified phosphoglycolipids [1].

## Genome sequencing and annotation

### Genome project history

This organism was selected for sequencing on the basis of its phylogenetic position, and is part of the *** G****enomic **** E****ncyclopedia of **** B****acteria and **** A****rchaea * project [13]. The genome project is deposited in the Genome OnLine Database [6] and the complete genome sequence is deposited in GenBank. Sequencing, finishing and annotation were performed by the DOE Joint Genome Institute (JGI). A summary of the project information is shown in Table 2.

**Table 2:** Genome sequencing project information

**MIGS ID**	**Property**	**Term**
MIGS-31	Finishing quality	Finished
MIGS-28	Libraries used	One Sanger libraries 8 kb pMCL200 and one 454 Pyrosequencing standard library
MIGS-29	Sequencing platforms	ABI3730, 454 GS FLX
MIGS-31.2	Sequencing coverage	9.2× Sanger, 26.9× Pyrosequencing
MIGS-30	Assemblers	Newbler, Arachne
MIGS-32	Gene calling method	Prodigal, GenePRIMP
	GenBank ID	CP001821 (chromosome), CP001822 (plasmid)
	GenBank Date of Release	November 20, 2009
	GOLD ID	Gc01153
	NCBI project ID	19715
	Database: IMG-GEBA	2501651194
MIGS-13	Source material identifier	DSM 15894
	Project relevance	Tree of Life, GEBA

### Growth conditions and DNA isolation

*X. cellulosilytica* XIL07^T^, DSM 15894, was grown in DSMZ medium 92 (Trypticase Soy Yeast Extract Medium) at 28°C [14]. DNA was isolated from 0.5-1 g of cell paste using Qiagen Genomic 500 DNA Kit (Qiagen, Hilden, Germany) following the manufacturer’s protocol without modifications.

### Genome sequencing and assembly

The genome was sequenced using a combination of Sanger and 454 sequencing platforms. All general aspects of library construction and sequencing performed at the JGI can be found at http://www.jgi.doe.gov/. 454 Pyrosequencing reads were assembled using the Newbler assembler version 1.1.02.15 (Roche). Large Newbler contigs were broken into 4,321 overlapping fragments of 1,000 bp and entered into assembly as pseudo-reads. The sequences were assigned quality scores based on Newbler consensus q-scores with modifications to account for overlap redundancy and to adjust inflated q-scores. A hybrid 454/Sanger assembly was made using Arachne assembler. Possible mis-assemblies were corrected and gaps between contigs were closed by custom primer walks from sub-clones or PCR products. Gaps between contigs were closed by editing in Consed, custom primer walk or PCR amplification. A total of 437 Sanger finishing reads were produced to close gaps, to resolve repetitive regions, and to raise the quality of the finished sequence. The error rate of the completed genome sequence is less than 1 in 100,000. Together all sequence types provided 36.1× coverage of the genome. The final assembly contains 52,128 Sanger and 514,866 Pyrosequencing reads.

### Genome annotation

Genes were identified using Prodigal [15] as part of the Oak Ridge National Laboratory genome annotation pipeline, followed by a round of manual curation using the JGI GenePRIMP pipeline [16]. The predicted CDSs were translated and used to search the National Center for Biotechnology Information (NCBI) nonredundant database, UniProt, TIGRFam, Pfam, PRIAM, KEGG, COG, and InterPro databases. Additional gene prediction analysis and manual functional annotation was performed within the Integrated Microbial Genomes Expert Review (IMG-ER) platform [17].

## Genome properties

**Figure 3:**
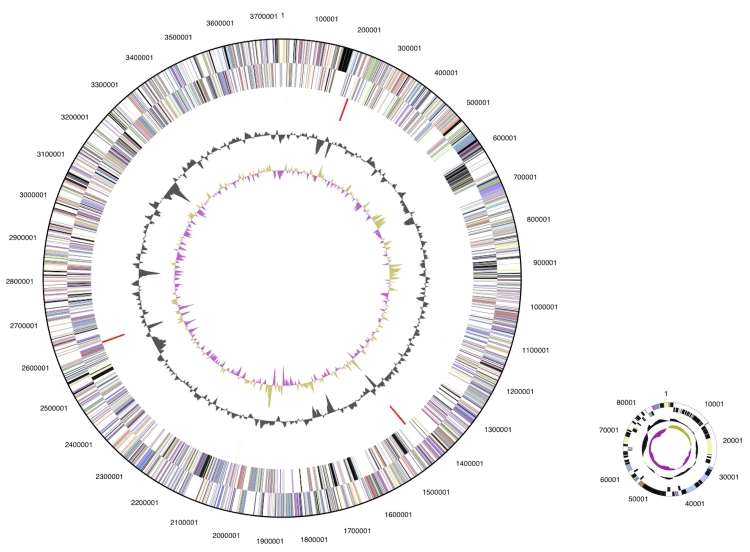
Graphical circular map of the genome. From outside to the center: Genes on forward strand (color by COG categories), Genes on reverse strand (color by COG categories), RNA genes (tRNAs green, rRNAs red, other RNAs black), GC content, GC skew.

The genome is 3,831,380 bp long and comprises one main circular chromosome and one plasmid with a 72.5% GC content (Table 3 and Figure 3). Of the 3,546 genes predicted, 3,485 were protein coding genes, and 61 RNAs. In addition, 42 pseudogenes were identified. The majority of the genes (68.4%) were assigned with a putative function while those remaining were annotated as hypothetical proteins. The distribution of genes into COGs functional categories is presented in Table 4.

**Table 3:** Genome Statistics

**Attribute**	**Value**	**% of Total**
Genome size (bp)	3,831,380	100%
DNA coding region (bp)	3,531,102	92.16%
DNA G+C content (bp)	2,775,913	72.45%
Number of replicons	2	
Extrachromosomal elements	1	
Total genes	3,546	100.00%
RNA genes	61	1.72%
rRNA operons	3	
Protein-coding genes	3,485	98.28%
Pseudo genes	42	1.18%
Genes with function prediction	2,426	68.42%
Genes in paralog clusters	411	11.59%
Genes assigned to COGs	2,403	67.77%
Genes assigned Pfam domains	2,490	70.22%
Genes with signal peptides	864	24.37%
Genes with transmembrane helices	925	26.09%
CRISPR repeats	1	

**Table 4:** Number of genes associated with the general COG functional categories

**Code**	**Value**	**%age**	**Description**
J	155	4.4	Translation, ribosomal structure and biogenesis
A	1	0.0	RNA processing and modification
K	243	7.0	Transcription
L	137	3.9	Replication, recombination and repair
B	1	0.0	Chromatin structure and dynamics
D	25	0.7	Cell cycle control, mitosis and meiosis
Y	0	0.0	Nuclear structure
V	85	2.4	Defense mechanisms
T	118	3.4	Signal transduction mechanisms
M	131	3.8	Cell wall/membrane biogenesis
N	6	0.2	Cell motility
Z	0	0.0	Cytoskeleton
W	0	0.0	Extracellular structures
U	39	1.1	Intracellular trafficking and secretion
O	77	2.2	Posttranslational modification, protein turnover, chaperones
C	153	4.4	Energy production and conversion
G	294	8.4	Carbohydrate transport and metabolism
E	214	6.1	Amino acid transport and metabolism
F	79	2.3	Nucleotide transport and metabolism
H	110	3.2	Coenzyme transport and metabolism
I	73	2.1	Lipid transport and metabolism
P	152	4.4	Inorganic ion transport and metabolism
Q	30	0.9	Secondary metabolites biosynthesis, transport and catabolism
R	318	9.1	General function prediction only
S	200	5.7	Function unknown
-	1082	31.0	Not in COGs
